# Marine heatwave and keystone predator loss drive broad‐scale decline and hinder recovery of a rocky intertidal kelp

**DOI:** 10.1002/eap.70215

**Published:** 2026-03-16

**Authors:** Francis D. Gerraty, Karah N. Cox‐Ammann, Melissa A. Douglas, Maya George, David P. Lohse, C. Melissa Miner, Peter T. Raimondi

**Affiliations:** ^1^ Department of Ecology and Evolutionary Biology University of California—Santa Cruz Santa Cruz California USA

**Keywords:** climate change, mussel, *Mytilus californianus*, *Pisaster ochraceus*, *Postelsia palmaeformis*, sea palm, sea star wasting

## Abstract

Human activities are increasingly driving the co‐occurrence of multiple ecological stressors, resulting in interactive and cumulative impacts that can reshape ecosystem dynamics and accelerate population declines of climate‐sensitive species. Here, we use over two decades of rocky intertidal monitoring data from 17 sites spanning over 1200 km of coastline to assess how two unprecedented stressors—a multiyear marine heatwave and the disease‐driven loss of a keystone predator (*Pisaster ochraceus*)—impacted populations of the canopy‐forming intertidal kelp *Postelsia palmaeformis.* We show that *Postelsia* experienced rapid and severe declines during the 2014–2016 northeast Pacific marine heatwave, with an average population decline of 50%, multiple site‐level extirpations, and particularly striking losses in the southern portion of the species' geographic range. Concurrently, *Pisaster* declines triggered mussel bed expansion into habitats previously occupied by *Postelsia*, further inhibiting kelp recoveries. Our findings reveal how converging stressors can drive persistent, broad‐scale ecological shifts through both direct and indirect pathways. These results also highlight the critical role of long‐term, spatially extensive monitoring in detecting and understanding global change impacts and provide a foundation for guiding *Postelsia* conservation and restoration efforts.

## INTRODUCTION

Climate change is increasing the frequency and magnitude of extreme climatic events, such as heatwaves, wildfires, droughts, and floods, leading to profound consequences for ecological systems and human wellbeing (Newman & Noy, 2023; Smith, 2011). These acute, punctuated events often interact synergistically with long‐term climatic changes or other stressors to drive extirpations, range shifts, and critical transitions from one ecosystem state to another. In the ocean, one of the most disruptive climate‐driven phenomena is the increasing prevalence of marine heatwaves (MHWs)—prolonged periods of anomalously high ocean temperatures (Marcos et al., 2025; Oliver et al., 2018; Wernberg et al., 2013). MHWs trigger direct physiological stress in marine organisms and can generate cascading indirect effects through altered species interactions (Smith et al., 2023). They have been linked to local and regional declines or extinctions of critical foundation species (Miner et al., 2021; Starko et al., 2022; Wernberg et al., 2024), ecosystem regime shifts (Rogers‐Bennett & Catton, 2019; Smith et al., 2024), and devastating social and economic consequences (Free et al., 2023; Smith et al., 2021). With MHWs becoming more frequent, prolonged, and intense, they are likely to continue compounding the effects of other unprecedented stressors to reshape ecological and evolutionary dynamics across global oceans (Oliver et al., 2018).

Infectious disease outbreaks are also a growing threat for wildlife and can produce dramatic disruptions to community dynamics and ecosystem function. While the emergence and spread of wildlife diseases is often facilitated by human activities such as habitat modification, global trade, and pollution, climate change is also altering host–pathogen dynamics in complex ways (Altizer et al., 2013; Burge et al., 2014; Mahon et al., 2024). In some cases, warming temperatures may reduce disease risk (Cohen et al., 2020), but more often shifting environmental conditions exacerbate pathogen impacts on wildlife (Harvell et al., 2002; Mahon et al., 2024). This is particularly concerning for foundation species and keystone predators, where disease outbreaks can initiate trophic cascades that ripple through food webs and restructure ecosystems (Buck & Ripple, 2017; Holdo et al., 2009, Moritsch, 2021). The intersection of warming temperatures and wildlife disease has already led to widespread mortality events in tropical and temperate oceans, with consequences for ecosystem services as well as human health and wellbeing (Burge et al., 2014).

Beginning in 2013, an invertebrate epizootic known as sea star wasting disease (SSWD) devastated intertidal and nearshore sea star populations along the northeast Pacific coast from Mexico to Alaska (Harvell et al., 2019; Miner et al., 2018). The disease spread rapidly in 2013–2015, affecting at least 20 asteroid species and causing severe declines in several critical predators including the sunflower sea star (*Pycnopodia helianthoides*) and the ochre star (*Pisaster ochraceus*, hereafter *Pisaster*) (Hamilton et al., 2021; Hewson et al., 2025; Konar et al., 2019; Menge et al., 2016; Miner et al., 2018). Immediately following the SSWD outbreak, an unprecedented multiyear MHW—known as the 2014–2016 northeast Pacific MHW—spanned from Baja California to the Gulf of Alaska and persisted from November 2013 to August 2016 in California, extending later at higher latitudes (Di Lorenzo & Mantua, 2016). This event, the longest recorded MHW to date, drove broad‐reaching ecological and social consequences including range shifts, mass mortality events, and fisheries closures (Cavole et al., 2016; Starko et al., 2025).

In northeast Pacific rocky subtidal and intertidal ecosystems, the confluence of the SSWD outbreak and the 2014–2016 MHW—two simultaneous and unprecedented stressors—has been associated with the collapse of subtidal kelp forests and expansion of urchin barrens (Burt et al., 2018; McPherson et al., 2021; Rogers‐Bennett & Catton, 2019; Schultz et al., 2016) as well as shifts from macrophyte‐ to invertebrate‐dominated intertidal communities (Meunier et al., 2024; Traiger et al., 2022; Whalen et al., 2023). The loss of *Pisaster*, a keystone predator that regulates rocky intertidal communities by preying on the California mussel (*Mytilus californianus*; Paine, 1966, Paine, 1974), likely played an impactful role in these intertidal regime shifts via trophic cascades (Meunier et al., 2024).

Despite the widespread ecological impacts of these simultaneous disturbance events, their effects on intertidal canopy‐forming kelps remain poorly understood. This is especially true for *Postelsia palmaeformis* (hereafter *Postelsia*), a culturally and economically important but critically understudied kelp that occupies wave‐exposed rocky intertidal shores (Ainis et al., 2019; Thompson et al., 2010; Turner, 2001). *Postelsia* has a patchy distribution (Dayton, 1973), extremely limited dispersal capacity (Coyer et al., 1997; Kusumo et al., 2006; Paine et al., 2017), and is sensitive to elevated water temperatures (Lüning & Freshwater, 1988; Muth et al., 2019; Young, 1971)—all traits that may heighten its vulnerability to climate change (Csordas et al., 2024). In addition, *Postelsia* commonly occurs among beds of competitively dominant *M*. *californianus* mussels (Dayton, 1973; Paine, 1979), potentially making the species vulnerable to mussel bed expansion triggered by *Pisaster* losses.

Here, we examine the direct and indirect effects of the 2014–2016 MHW and the synergistic SSWD outbreak on the abundance, persistence, and recovery of *Postelsia*. To do so, we leverage long‐term rocky intertidal community monitoring data collected for over two decades at 17 sites spanning most of the southern portion of the kelp's geographic range. We hypothesized that *Postelsia* declined due to heat stress during the 2014–2016 MHW, and that *Pisaster* loss led to mussel bed expansion, indirectly hindering *Postelsia* recovery (Meunier et al., 2024). Consequently, we predicted that these synergistic disturbances acted as a “one‐two punch” to drive persistent, broad‐scale *Postelsia* declines.

## MATERIALS AND METHODS

### Study system

**FIGURE 1:**
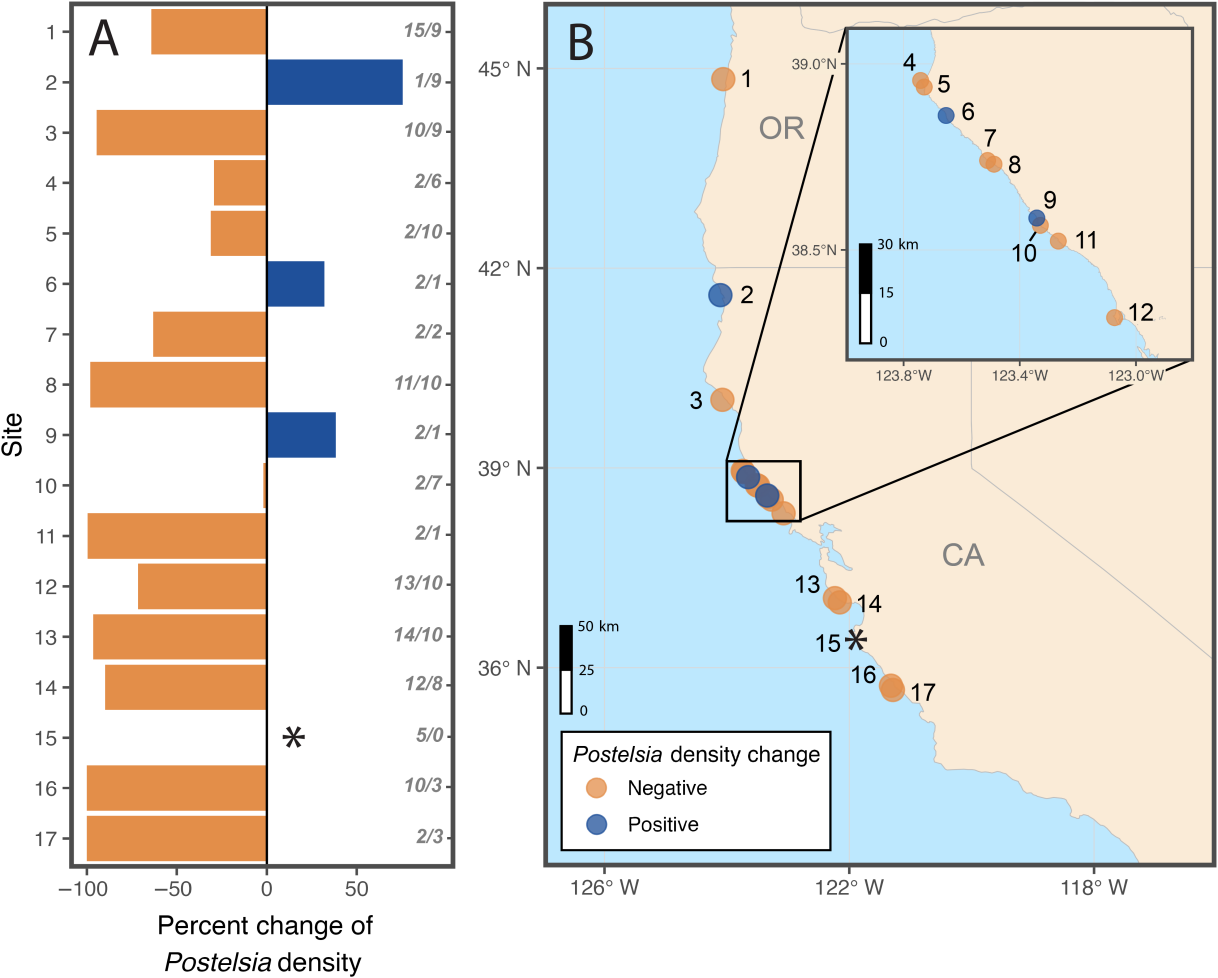
(A) Patterns of change in *Postelsia* density associated with the 2014–2016 northeast Pacific marine heatwave (MHW). Bars display the change in mean densities at each site between *Postelsia* surveys before (≤2014) versus during and after (≥2015) the MHW. Gray, italicized numbers indicate the number of *Postelsia* surveys before/during and after the MHW. (B) Locations of *Postelsia* long‐term monitoring sites in California and Oregon. Orange bars (A) and circles (B) indicate post‐MHW declines in *Postelsia* density and blue bars/circles indicate post‐MHW density increases. *No *Postelsia* surveys were conducted post‐MHW at one site and density changes could therefore not be determined.

We studied rocky intertidal communities at 17 sites that were established as part of the Multi‐Agency Rocky Intertidal Network (MARINe, www.pacificrockyintertidal.org), a collaborative long‐term monitoring program designed to capture natural dynamics and patterns of change in rocky intertidal communities along the Pacific Coast of North America. Our study region, spanning over 1200 km of coastline from Central Oregon to Central California, USA (Figure 1), encompasses over half of the latitudinal distribution of *Postelsia* and includes the vast majority of the species' equatorial “trailing edge” of its geographic range. This annual brown alga, colloquially known as “sea palm,” occurs along wave‐exposed rocky intertidal shores from Central California, USA, to Cranston Point, British Columbia, Canada (Abbott & Hollenberg, 1976; Z. Monteith, personal communication).

*Postelsia* exhibits a patchy distribution at both broad and local spatial scales, with presence tightly linked to areas of extreme wave shock (Dayton, 1973). This canopy‐forming species typically co‐occurs in the mid‐tidal zone with the mussel *Mytilus californianus*, but in areas of high wave exposure its tidal distribution can extend into the high zone (Dayton, 1973; Paine, 1979). Due to its life cycle, the sporophytic stage of *Postelsia—*the stage capable of defending space on the rock against mussel encroachment—is not present for several months each year (Paine, 1979). Thus, successful recruitment of successive generations of sporophytes often depends on wave‐induced disturbances (Blanchette, 1996; Dayton, 1973; Paine, 1979; Paine, 1988) and predation by *Pisaster* sea stars to open up space for colonization within mussel beds (Dayton, 1971; Paine, 1966; Paine, 1974). However, while mussels will ultimately outcompete and exclude *Postelsia* in the absence of disturbance, mussel beds may also provide a protective microhabitat that enhances the survival of microscopic stages of *Postelsia* during the fall and winter months (Blanchette, 1996).

Spores generally settle within 2 m from their parent plant, and patches >25 m apart typically show distinct genetic structure (Coyer et al., 1997; Kusumo et al., 2006; Paine et al., 2017). Long‐distance dispersal events are thought to be exceedingly rare, limited to occasional floating individuals that have been ripped from the substratum (Paine et al., 2017). Thus, if *Postelsia* is locally extirpated from an area, as was observed for a population at the southern edge of the alga's range during the 1982–1983 El Niño‐Southern Oscillation (ENSO) event (Young, 1971; J. Steinbeck, personal communication), natural recovery might never occur.

High habitat specificity, poor dispersal ability, and sensitivity to warm water temperatures, combined with its inferior competitive status with mussels, might make *Postelsia* particularly vulnerable to the combined effects of MHWs and keystone predator losses. However, apart from a recent investigation near the species' leading (cold‐water) range edge (Vancouver Island, BC) that found long‐term stability of *Postelsia* from 2006 to 2022 (Csordas et al., 2024), the impacts of these paired disturbances on *Postelsia* populations remains unknown. Assessing potential impacts in the southern portion of the species' range is critical because disturbance is likely to be greater in this region, where warmer water temperatures might lead to greater acute physiological stress on *Postelsia*. In addition, reduced wave exposure in the south might lower the tidal height distribution of *Postelsia*, thereby increasing spatial overlap with mussels and *Pisaster* and making the kelp more vulnerable to the indirect effects of *Pisaster* declines.

### *Postelsia* surveys

To monitor long‐term changes in *Postelsia* abundance across a broad geographic scale, we established 1–3 permanent transects at each of 17 MARINe sites along the California and Oregon coastline (Figure 1). Starting in 1999, fixed plots were established in areas where *Postelsia* was initially abundant and could be accessed safely. These plots typically consisted of 2‐m‐wide band transects delimited by bolts anchored to the substratum and varying in length from 5 to 20 m. We counted the total number of *Postelsia* within each band transect during each survey. To avoid issues with seasonality due to the annual life history of *Postelsia*, sites were generally sampled in the same period (i.e., a “canonical season”) each year (March–April in Central California, May–June in Northern California, and July in Oregon). Some sites were surveyed in noncanonical seasons or multiple seasons in some years; in these cases, we only included surveys conducted in the site's canonical season for analyses.

**FIGURE 2:**
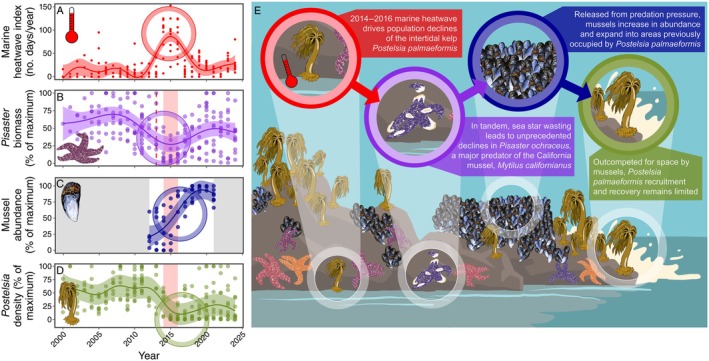
Progression of events affecting *Postelsia* abundance and temporal changes in the abundance of *Pisaster*, mussels (*M*. *californianus*), and *Postelsia*. (A) Total number of marine heatwave (MHW) days per year at each site with intertidal water temperature loggers (including all heatwave categories), (B) *Pisaster* biomass in sea star monitoring plots, (C) mussel percent cover from panoramic photos at *Postelsia* transects, which were only digitized from 2012 to 2021, and (D) *Postelsia* abundance—all modeled with generalized additive mixed‐effects model with year as a smoothing term and site (or transect for mussels) as a random effect. Shaded pink region in panels (B–D) represents the 2014–2016 MHW and the red, vertical dashed line in panel (B) represents the onset of the SSWD outbreak. (E) Key events and ecological factors influencing *Postelsia* abundance. Illustration credit: Francis D Gerraty.

To determine temporal dynamics, we first identified the year with the highest recorded *Postelsia* density at each site and expressed all other survey estimates at the site as a percentage of this maximum density. We then visualized mean annual trends in *Postelsia* density with a generalized additive mixed‐effects model fit to these percent of maximum density values, with year as a smoothing term and site as a random effect (Figure 2D). The model was fit using restricted maximum likelihood (REML) using the gam function in the mgcv package in R (version 4.5.0; Wood, 2011, R Core Team, 2025). We then directly compared mean *Postelsia* densities between periods before the MHW (≤2014) versus during and after the MHW (≥2015) to determine site‐level and regional metrics of MHW population impact (Figure 1A). To assess population recoveries, we also compared mean *Postelsia* densities among three periods, before the MHW (≤2014), during the MHW (2015–2016), and after the MHW (≥2017), with a generalized linear mixed‐effects model with a tweedie distribution, log link function, and site as a random effect. The model was fit using the glmmTMB function in the glmmTMB package, and assumptions were checked using the DHARMa package (Brooks et al., 2017, Hartig, 2022).

### Water temperature

**FIGURE 3:**
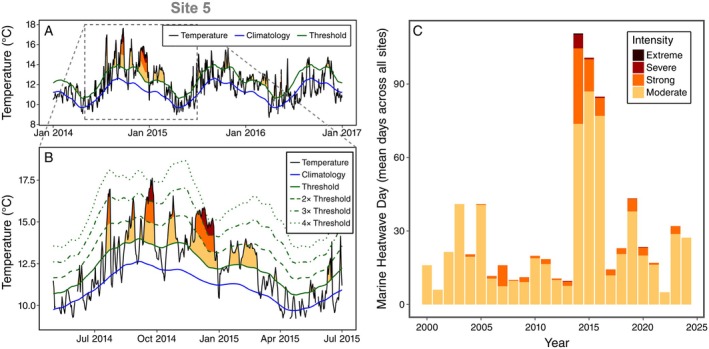
Intertidal water temperatures measured in rocky intertidal zones during the 2014–2016 marine heatwave (MHW). (A) Water temperature at one site (site 5) throughout the MHW. When water temperatures (black line) exceeded the 90th percentile (green solid line) relative to the local long‐term climatology (blue line) for five or more days, then MHW events were detected (colored fills). MHW events were categorized based on how much the temperature exceeded this threshold (moderate = 1–2 ×, strong = 2–3 ×, severe = 3–4 ×, extreme = >4 ×). Panel (B) shows these categorical thresholds and provides detail of the most extreme portion of the MHW at site 5. (C) Mean number of days per year across all sites that fell within each MHW category.

As part of the MARINe program, we measured intertidal water temperatures at 10 of the *Postelsia* monitoring sites (sites 1–3, 5, 8, 12–14, 16, and 17) using HOBO Pendant and TidbiT (v1 and v2) temperature loggers (Onset Computer Corporation, Bourne, Massachusetts, United States) housed in stainless steel mesh cages anchored to the substratum at the interface between middle and low intertidal zones, beginning in 2000 at the earliest sites. After downloading logger data, we extracted water temperatures on the basis of daily tidal cycles and removed any outlier temperatures. We calculated daily mean water temperature for each site by averaging all water temperature measurements, then determined the baseline temperature climatology and detected MHW events at each site using the HeatwaveR package (Schlegel & Smit, 2018). Following Hobday et al., 2016, MHWs were defined as warm water periods lasting 5 days or more above the 90th percentile relative to long‐term, period‐specific local climatology and were categorized from moderate (Category I) to extreme (Category IV) based on heatwave intensity (Figure 3). Categories were based on multiples of the value represented by the difference between the local climatological mean and the climatological 90th percentile, with categories defined as “moderate” (Category I; 1–2 × the local difference), “strong” (Category II; 2–3 ×), “severe” (Category III; 3–4 ×), and “extreme” (Category IV; >4 ×) (Hobday et al., 2018).

We calculated the number of days per year within each heatwave category at all sites (Appendix S1: Figure S1), and the mean number of days per year in each heatwave category across all sites (Figure 3C). Finally, we determined the total number of MHW days per year at each site (including all heatwave categories) and fitted a generalized additive mixed‐effects model to these values with year as a smoothing term and site as a random effect using REML to visualize temporal trends (Figure 2A). There were temporal gaps in water temperature data at several sites due to temperature logger failure, malfunction, and loss (e.g., Appendix S1: Figures S1 and S2), so we removed all sites with over 25 missing days of temperature monitoring in any given year prior to multisite data summarization.

### Repeated photos

**FIGURE 4:**
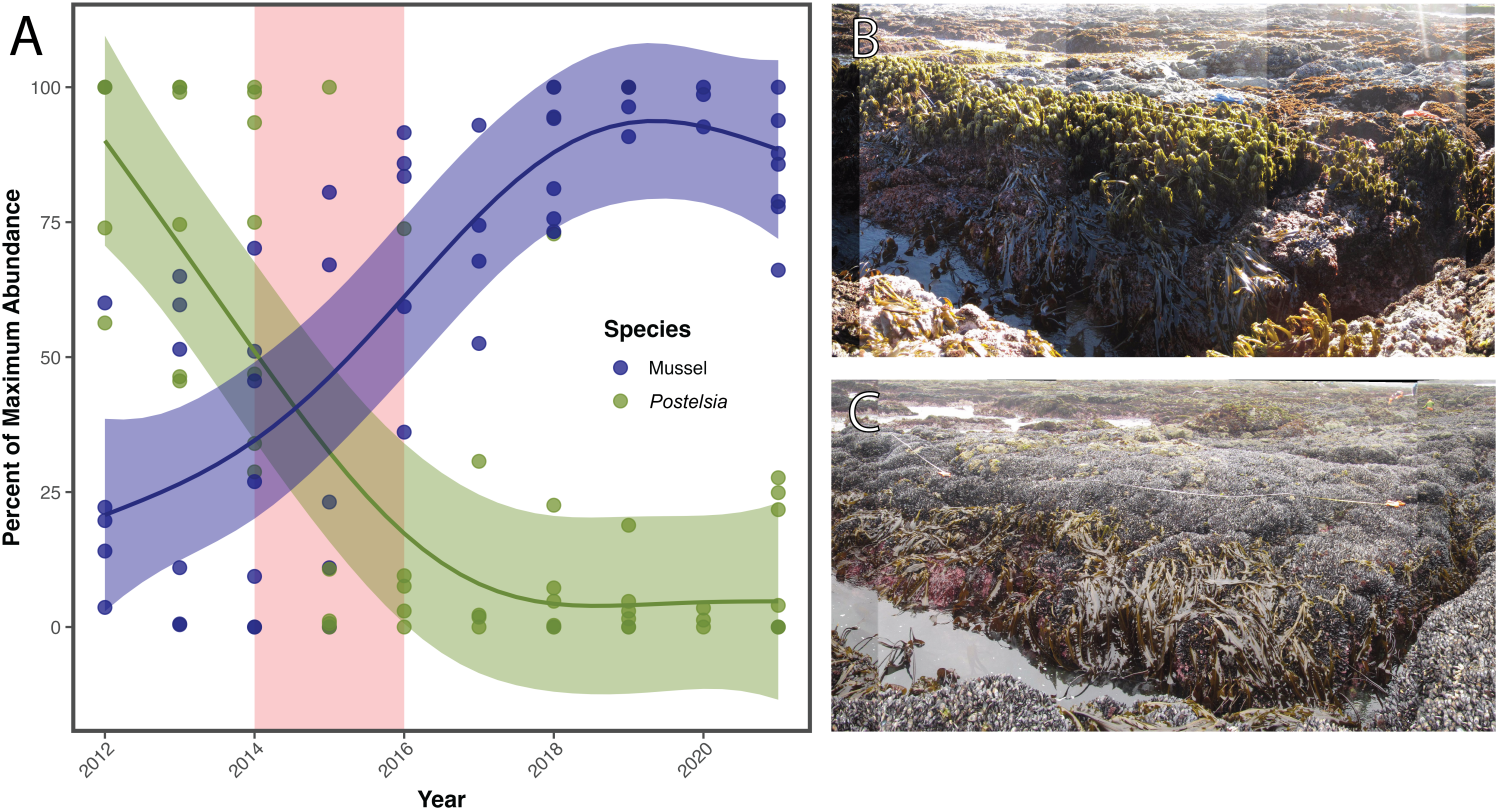
(A) Temporal changes in the abundance (maximum percent cover within standardized box) of *Postelsia* and mussels (*M*. *californianus*) in repeated panoramic photos. *Postelsia* and mussel cover in each photo was modeled with a generalized additive mixed‐effects model (for each species) with year as a smoothing term and transect as a random effect. Photographic examples of panoramic photos at site 3 before (B) and after (C) the 2014–2016 marine heatwaves. Photos in (B) and (C) courtesy of the Multi‐Agency Rocky Intertidal Network.

At five sites (sites 3, 8, 12, 13, and 16), the abundance of *Postelsia* and mussels were quantified using panoramic photos taken from fixed locations adjacent to *Postelsia* transects (two repeat panoramic photos at two sites, one panoramic photo at three sites). Using Adobe Photoshop, a standardized box was drawn around the same location in each image, and *Postelsia* and mussel patches were manually outlined using heads‐up digitization (i.e., digitally tracing features on the image) to determine percent cover within the box. We digitized 6–10 panoramic photos from each site (55 photos total) taken between 2012 and 2021. To visualize temporal shifts in *Postelsia* and mussel percent cover within these photos, we calculated the percentage of maximum abundance (maximum percent cover within box) for each photo, then fitted a generalized additive mixed‐effects model for each species with year as a smoothing term and transect as a random effect using REML (Figure 4).

### Sea star and mussel surveys

Beginning in 2000, ochre stars (*Pisaster*) were counted and measured at 15 sites (sites 1–13, 16, and 17) within three irregularly shaped plots (20–160 m^2^) permanently established in areas of initially high *Pisaster* density (Miner et al., 2018). The “radius” of each *Pisaster* was measured as the distance from the center of the star to the tip of its longest ray, estimated to the nearest 10 mm for individuals >7 mm. Stars 3–7 mm were grouped into a 5‐mm radius bin, and those <3 mm were not counted due to inconsistent detection.

To standardize sea star abundance estimates across years, we calculated *Pisaster* biomass at each site using the length‐biomass conversion equation from Moritsch and Raimondi (2018). We converted counts and size structure to biomass because predator biomass is a more ecologically relevant proxy for predation pressure. We then identified the year with the highest recorded biomass at each site and expressed all other survey estimates as a percentage of this maximum. While this approach does not allow for direct comparisons of *Pisaster* density or biomass between sites due to plot irregularities, it enables comparisons of relative biomass trends within each site over time. To visualize *Pisaster* declines associated with SSWD, we fit a generalized additive mixed‐effects model to the relative biomass estimates, using year as a smoothing term and site as a random effect using REML (Figure 2B). We then compared these relative biomass estimates among periods before the SSWD outbreak (≤2013), during the SSWD outbreak (2013–2016), and after the SSWD outbreak (≥2017) using a linear mixed‐effects model with site as a random effect. The model was fit using the lmer function in the lme4 package, and assumptions were checked using the DHARMa package (Bates et al., 2015; Hartig, 2022).

To document shifts in mussel abundance associated with *Pisaster* loss at a larger scale than the repeated photos dataset described above, we used data from MARINe Coastal Biodiversity Surveys (CBS), conducted from 2001 to 2023, to compare mussel percent cover and tidal height distribution among surveys before and after the SSWD outbreak. Of the 17 sites, only 13 had CBS surveys both before (≤2013) and after (≥2014) the SSWD outbreak; analyses were therefore limited to these sites (1, 3, 4, 6, 7–10, 12–14, 16, and 17). CBS are designed to capture the abundance and distribution of all common taxa, including mussels, across the intertidal zone at fixed locations within each site, allowing for assessment of change through time. A fixed, uniformly distributed grid was established at each site, consisting of 11 vertical parallel transect lines spaced 3 m apart, with approximately 100 points per transect distributed across the full tidal range, which resulted in approximately 1100 points per site. Organisms under each point were identified to the lowest possible taxonomic level, including layering and epibionts when appropriate (see https://www.pacificrockyintertidal.org for full description of methods).

The upper elevational limit of mussels is restricted by temperature and desiccation, whereas the lower limit is regulated by *Pisaster* predation (Dayton, 1971; Paine, 1974). Because of this, we hypothesized that SSWD‐driven *Pisaster* declines would cause mussels to (1) increase in percent cover across the intertidal and (2) expand to lower tidal heights at the lower edge of its vertical distribution. To assess changes in mussel percent cover between the periods before and after SSWD (≤2013 vs. 2014–2023; hypothesis 1), we determined the percent cover of *M. californianus* in each CBS survey (number of *M. californianus* points/all survey points × 100) and then fit a linear mixed‐effects model with time period (pre/post SSWD) as a fixed effect and site as a random effect to these percent cover values. This model was fit using the lmer function in the lme4 package, and assumptions were checked using the DHARMa package (Bates et al., 2015, Hartig, 2022). To assess shifts in the tidal height of the lower distributional limit of *M. californianus* before and after the SSWD outbreak, we first determined the tidal height of each mussel detection relative to mean lower low water (MLLW). This was done by integrating biological survey data with transect‐level topographic profiles obtained using a rotating laser level. We then fit linear quantile mixed‐effects models based on the asymmetric Laplace distribution, with time period (pre/post SSWD) as a fixed effect and transect as a random effect, to three quantiles of mussel tidal height distributions (τ = 0.1, 0.5, and 0.9; i.e., 10th, 50th, and 90th percentiles) using the lqmm function in the lqmm package (Geraci, 2014; Geraci & Bottai, 2014). We used quantile regression because it allowed us to assess changes across the full distribution of mussel tidal heights—not just the mean—thereby capturing shifts in both the core and boundaries of the species' vertical range.

Importantly, permanent sea star monitoring plots and CBS grid locations do not always directly overlap with *Postelsia* permanent transects, but they are typically within 50 m and always within 200 m of one another. To assess tidal height overlap among *Pisaster*, *M. californianus*, and *Postelsia*—thereby providing additional evidence that mussel predation by *Pisaster* may facilitate *Postelsia* habitat formation—we extracted CBS grid data from all MARINe surveys in which the three species co‐occurred. This yielded tidal height distributions for each species from 44 CBS surveys conducted between 2001 and 2023 across 13 MARINe sites, which we visualized at the site level using kernel density estimation (Appendix S1: Figure S3).

### Statistical analysis and visualization software

All analyses were conducted in R version 4.5.0 (R Core Team, 2025) using the following packages: tidyverse (v2.0.0, Wickham et al., 2019), heatwaveR (v0.5.4, Schlegel & Smit, 2018), mgcv (v1.9.1, Wood, 2011), glmmTMB (v1.1.11, Brooks et al., 2017), DHARMa (v0.4.7, Hartig, 2022), lme4 (v1.1‐37, Bates et al., 2015), and lmqq (v1.5.8, Geraci, 2014, Geraci & Bottai, 2014). Figures and tables were generated using ggplot2 (v3.5.2, Wickham, 2016), ggthemes (v5.1.0, Arnold, 2021), sf (v1.0.20, Pebesma, 2018), maps (v3.4.2.1, Becker et al., 2024), gratia (v0.10.0, Simpson, 2024), gt (v1.0.0, Iannone et al., 2025), and biscale (v1.0.0, Prener et al., 2022). Data and code to reproduce all analyses and figures are publicly available (Gerraty et al., 2026).

## RESULTS

**FIGURE 5:**
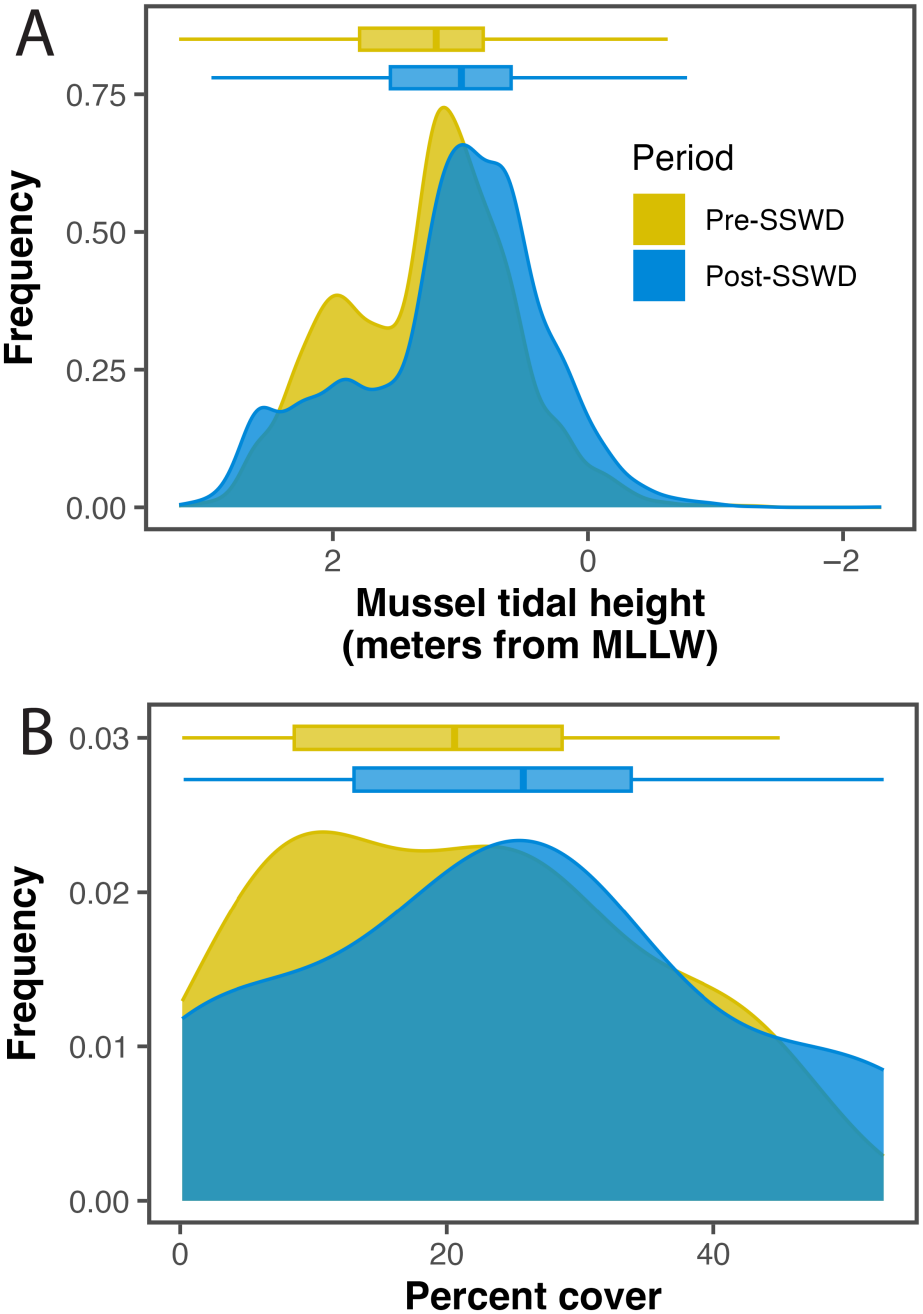
Changes in mussel (*M. californianus*) tidal height distribution (A) and frequency of percent cover at the site‐wide scale (B) before (≤2013) and after (≥2014) the sea star wasting disease (SSWD) outbreak. MLLW, mean lower low water.

We found evidence that the 2014–2016 MHW initiated severe, broad‐scale *Postelsia* declines and that the synergistic SSWD outbreak indirectly hindered *Postelsia* recoveries (Figure 2). *Postelsia* abundance declined rapidly in 2014–2015 during the onset of the MHW, leading to local extirpations at the two southernmost sites (sites 16 and 17) and substantially reduced abundance in most other sites (Figure 1, Figure 2). Beginning in 2013, the SSWD outbreak led to sharp declines of *Pisaster*—a keystone predator—which were associated with an increase in mussel (*M. californianus*) abundance, mussel expansion to lower tidal heights, and mussel colonization of habitats formerly occupied by *Postelsia* (Figure 2, Figure 4, Figure 5).

### Direct effects of MHW

The northeast Pacific MHW began during summer 2014 and persisted through autumn 2016 in our study region. Although subregional variation was evident, the most intense period—based on temperature anomalies and the number of MHW days—occurred from September 2014 to April 2015 (Figure 3; Appendix S1: Figures S1 and S2; Gentemann et al., 2017). After April 2015, water temperatures cooled in the northern portion of the study area, while the southern portion experienced another severe temperature spike from August to October 2015. Across sites with continuous temperature monitoring (<25 days of temperature data missing per year), the mean number of MHW days was 110.6 ± 10.5 (SD) in 2014 and 100.8 ± 25.9 (SD) in 2015 (Figure 3C). Nine of 10 sites with water temperature loggers (excluding site 1) recorded maximum water temperatures exceeding 17°C (Appendix S1: Table S1). Of these, five sites (sites 12–14, 16, 17) had maximum temperatures that exceeded 18°C, and one site (site 14) reached 20°C (Appendix S1: Table S1). Most MHW events were classified as “moderate,” but “strong” and “severe” events occurred at multiple sites in both 2014 and 2015 (Figure 3). One site (site 5) experienced an “extreme” MHW event in 2014, exceeding 4 × the MHW threshold (Figure 3; Appendix S1: Table S1).

*Postelsia* densities declined after the onset of the MHW at 13 out of 16 sites (81%) where pre‐MHW (≤2014) and post‐MHW (≥ 2015) surveys were conducted (Figure 1, Appendix S1: Table S2). Across all sites, *Postelsia* densities declined by a mean of 49.6% between these periods. These declines often occurred rapidly (i.e., within 1–2 years) after MHW onset, suggesting that anomalously warm water temperatures likely triggered direct physiological stress (Appendix S1: Figure S4; Young, 1971; Lüning & Freshwater, 1988).

Declines in *Postelsia* were particularly pronounced in the southern portion of our study region; all sites south of Salmon Creek, Sonoma County (sites 11–17), exhibited >70% declines in density and local extirpations occurred at the two southernmost sites in 2015 (sites 16 and 17; Figure 1). Another site (site 13) experienced a local extirpation in 2016, but the population began to recover in 2018–2024, albeit to drastically lower densities than those documented prior to the MHW (Appendix S1: Figure S4). Changes in *Postelsia* density were more variable north of Salmon Creek (sites 1–10), ranging from two sites with >90% declines to one site with a 75.5% increase in density (Figure 1; Appendix S1: Table S2). However, the three sites with post‐MHW density increases had limited sampling—only 1–2 surveys before the MHW, and just one survey after the MHW at two of three sites. Low sampling effort at these sites may have influenced our density change estimates due to stochastic variation (Appendix S1: Figure S5).

Our generalized linear mixed‐effects model comparing *Postelsia* density before (≤2014), during (2015–2016), and after (≥2017) the MHW provided strong evidence that density differed significantly among all time periods. *Postelsia* density was highest prior to the MHW (estimated marginal mean: 56.47 ± 7.09 SE individuals/m^2^), declined sharply during the MHW (3.58 ± 1.02 SE), and partially rebounded post‐MHW (12.09 ± 1.78 SE).

### Indirect effects of sea star wasting

Sea star wasting was first documented in our study region in 2013, leading to rapid, significant declines in *Pisaster* biomass at all sites with high monitoring frequency except for site 8 (Miner et al., 2018; Figure 2B; Appendix S1: Figure S6). Prior to the SSWD outbreak, *Pisaster* mean biomass was 60.4% ± 3.91 SE (estimated marginal mean) of the maximum biomass recorded in the sea star monitoring plots. In 2013–2016, after the onset of SSWD, *Pisaster* mean biomass collapsed to only 26.4% ± 5.34 SE of the maximum documented biomass. *Pisaster* populations began to rebound at most sites after 2016, although *Pisaster* biomass at a few—including the two southernmost sites—remain well below pre‐SSWD levels (i.e., see sites 3, 16, and 17 in Appendix S1: Figure S6). After 2016 (i.e., 2017–2024), *Pisaster* rebounded to 45.2% ± 3.85 SE of the maximum documented biomass. Importantly, biomass recovery does not necessarily mean the recovery of keystone predation pressure, particularly because a periodic absence of predation may allow mussels to grow beyond the size at which *Pisaster* can consume them (Paine, 1976).

We found that *Pisaster* declines were temporally associated with increases in mussel abundance and mussel bed expansion to lower tidal heights. We conducted 51 CBS surveys at 13 sites between 2001 and 2023 to detect shifts in mussel abundance and distribution. Of these, 26 surveys occurred before the SSWD outbreak onset (≤2013), and 25 occurred after. The frequency and timing of CBS surveys varied among sites; for example, site 9 had only one survey before and one after the SSWD outbreak, whereas site 12 had five surveys before and three after. Across all CBS surveys, mussels (*M. californianus*) were detected at 12,499 survey points. Mussel percent cover increased significantly after the SSWD outbreak (estimate = 7.51% ± 1.05 SE, *p* < 0.001). Quantile regression revealed a significant lowering in the tidal height of the 10th and 50th (i.e., lower elevation and median) percentiles of mussel distribution after the SSWD outbreak, with no significant change at the 90th percentile. At the 10th percentile (τ = 0.1), mussels occurred significantly lower in the intertidal zone (estimate = −0.13 m ± 0.04 SE, *p* < 0.001), indicating a downward shift in the lower bounds of their vertical distribution. A similar but slightly smaller downward shift was observed at the median (estimate = −0.11 m ± 0.04 SE, *p* = 0.012). As expected, no significant change was detected at the 90th percentile (estimate = −0.03 ± 0.03 m SE, *p* = 0.304), suggesting that the upper tidal limit of mussels remained relatively stable following *Pisaster* declines. CBS grid data from surveys in which *Pisaster*, *M. californianus*, and *Postelsia* co‐occurred revealed substantial overlap in their tidal height distributions at nearly all sites, but did not show any latitudinal differences in *Postelsia* tidal height distribution (Appendix S1: Figure S3).

Although sea star monitoring and CBS surveys provided compelling evidence that *Pisaster* declines were linked to increases in mussel abundance and mussel bed expansion to lower tidal heights, mechanistic detail on how these ecological shifts impacted *Postelsia* recoveries is limited due to the lack of direct overlap among sea star monitoring plots, CBS survey grids, and *Postelsia* transects. However, our repeated panoramic photos provided complementary insights into how *Postelsia*‐mussel interactions shifted during and after the MHW and SSWD outbreak. In 2012–2013, immediately prior to the MHW and SSWD outbreak, *Postelsia* mean cover was 81.4% ± 6.9 SE of the maximum percent cover in the repeated photos. By contrast, mean mussel cover was 28% ± 7.7 SE of its maximum percent cover. However, a dramatic shift occurred from 2014 to 2016, after which *Postelsia* mean cover dropped to 9.4% ± 3.1 SE of its maximum abundance and mussel mean cover expanded to 88% ± 2.5 SE of its maximum abundance (Figure 4; Appendix S1: Figure S8). These data reveal that mussel beds expanded into areas previously occupied by *Postelsia* after the MHW and SSWD outbreak, a trend that coincided with sharp declines in *Postelsia* cover (Figure 4).

## DISCUSSION

Our study highlights how multiple, concurrent disturbance events can reshape population and community dynamics in strong and complex ways. We show that the 2014–2016 MHW was correlated with rapid and widespread *Postelsia* declines and that disease‐driven *Pisaster* losses likely hindered kelp persistence and recovery by releasing competitively dominant mussels from keystone predation pressure (Figure 2). These results underscore the importance of considering both direct and indirect pathways when evaluating global change impacts on climate‐sensitive species. Our findings also emphasize the critical role of long‐term monitoring for detecting and understanding climate impacts and provide a foundation for guiding *Postelsia* conservation and management efforts.

### Converging stressors

Ecological impacts of disturbance events tend to be studied in isolation, largely because acute perturbations have historically been rare, but global climate change and other anthropogenic stressors are increasing both the frequency and temporal overlap of extreme disturbances (Buma, 2015; Newman & Noy, 2023; Smith, 2011). Our findings suggest that two simultaneous and unprecedented disturbance events—a MHW and a keystone predator disease outbreak—negatively impacted *Postelsia* through different mechanisms: the MHW caused direct physiological stress, while *Pisaster* declines indirectly limited kelp persistence and recovery by enabling the expansion of competitively dominant mussels into *Postelsia* habitats (Figure 2). While attribution of change is challenging during simultaneous disturbance events, it is likely that the population‐level impacts on *Postelsia* were more severe than would be expected if each disturbance had occurred independently. Forecasting long‐term population dynamics for climate‐sensitive species must therefore account for interactive and compounding effects of simultaneous disturbance events, such as those documented here (Buma, 2015).

Warm water temperatures have long been considered an important determinant of the southern distributional edge of *Postelsia*, despite an absence of field studies in the region documenting such associations (Lüning & Freshwater, 1988; Young, 1971). Laboratory studies have documented that *Postelsia* sporophytes are unable to withstand prolonged water temperatures >15°C, a value that roughly corresponds with the average maximum summer temperature at the southern range edge in San Luis Obispo county (Lüning & Freshwater, 1988). In addition, Muth et al. (2019) found that *Postelsia* exhibited recruitment failure in a laboratory setting at 18°C. Every site with temperature loggers had more than 70 days where mean water temperatures exceeded 15°C, and the southernmost five of these sites had multiple days where water temperatures exceeded 18°C (Appendix S1: Table S1). While lab‐based thermal tolerance studies must be interpreted with caution because *Postelsia* likely exhibits local thermal adaptation due to its extremely limited dispersal, our findings are consistent with and build upon these results to suggest that climate‐driven range retraction at the southern range edge of *Postelsia* is likely underway. This pattern is also consistent with widespread reports of kelp range contractions at warm range limits globally (Smale, 2020).

Identifying the interactive and cumulative impacts of both acute MHWs and chronic ocean warming on the distribution of *Postelsia* remains a critical research gap. A glimpse of historical insight is provided by Young (1971), which documented substantial *Postelsia* patches in one location at or near the kelp's southern range limit. However, all nearby patches were extirpated during the 1982–1983 ENSO event and have not recovered (J. Steinbeck, personal communication). Documenting fine‐scale distributional shifts and critical persistence thresholds, and also identifying the ecological and social consequences of *Postelsia* losses, are important research directions that can inform conservation and management strategies.

Our research adds to and is reinforced by a broad scientific understanding surrounding the negative impacts of MHWs on kelps in both subtidal and intertidal systems (Eger et al., 2024, Roethler et al., 2025, Wernberg et al., 2024, Weitzman et al., 2021, Whalen et al., 2023). However, the cascading ecological consequences of disease outbreaks in predators have been less comprehensively documented, especially in marine ecosystems (Buck & Ripple, 2017; Burge et al., 2014; Schultz et al., 2016). Our findings highlight how keystone predator losses can cascade through food webs to reshape population trajectories of climate‐sensitive species. *Pisaster* losses were followed by increasing mussel abundance and mussel expansion to lower tidal heights, a result consistent with other studies documenting shifts from macroalgal to invertebrate‐dominated intertidal communities during the SSWD outbreak and 2014–2016 MHW (Meunier et al., 2024; Traiger et al., 2022; Weitzman et al., 2021; Whalen et al., 2023). Mussels are a dominant competitor for space and can strongly influence rocky intertidal biodiversity and community composition (Miner et al., 2021; Paine, 1974), so the negative ramifications of mussel bed expansion for *Poselsia* persistence and recovery are likely just one of numerous ecological consequences arising in the aftermath of the SSWD outbreak. We encourage future research efforts to trace the diverse ecological legacies of SSWD‐triggered *Pisaster* losses and explore how they continue to reshape coastal ecosystem dynamics into the present (Meunier et al., 2024; Smith et al., 2025).

While our results are broadly consistent with *Pisaster* functioning as a keystone predator in maintaining habitat heterogeneity and influencing *Postelsia* population trajectories, the magnitude and nature of these predator effects are not uniform across space and time (Menge et al., 1994; Menge et al., 2021). The indirect effects of *Pisaster* losses on *Postelsia* recoveries are contingent on the overlapping tidal height distributions of *Pisaster*, mussels, and *Postelsia*—which we documented across our study region (Appendix S1: Figure S3)—and are likely strongest at sites with high mussel abundance. However, because these keystone interactions depend on local conditions such as wave exposure and mussel recruitment variability, the indirect effects of *Pisaster* abundance on *Postelsia* population dynamics likely vary among regions and ecological contexts. For example, greater wave exposure at northern latitudes might extend the vertical distribution of *Postelsia* into the high intertidal zone (as described by Dayton, 1973 and Paine, 1988), thus reducing spatial overlap among *Pisaster*, mussels, and *Postelsia*, and thereby weakening the influence of *Pisaster* on *Postelsia* habitat formation. In addition, the importance of *Pisaster* for creating *Postelsia* settlement habitat might be greatly reduced at northern latitudes, where wave disturbance—often in the form of log‐bashing—is a widely documented source of mussel removal (Dayton, 1973; Paine, 1988). Indeed, Csordas et al. (2024) reported stable *Postelsia* populations in British Columbia over the same period as our study.

### Implications for conservation and management

Our findings of widespread and persistent *Postelsia* declines have direct implications for the management of this ecologically, economically, and culturally significant kelp. *Postelsia* is currently harvested for both commercial and recreational purposes, with regulations varying across its range depending on state or provincial jurisdiction. The most common harvesting practice is to trim blades above the meristem, which allows individuals to survive and blades to regrow, but this method can still reduce spore production and hinder recruitment (Thompson et al., 2010). Blade harvest after the onset of sporogenesis has been shown to sharply reduce spore production, suggesting that harvest timing is critical for sustainability of the *Postelsia* fishery (Thompson et al., 2010). To mitigate detrimental harvest impacts, potential management actions include mandating blade‐trimming harvest methods, restricting harvest to a defined season that ends prior to the onset of sporogenesis, and establishing temporary or permanent closures for both recreational and commercial take in areas experiencing decline or where remnant populations are failing to recover (Thompson et al., 2010).

Because *Postelsia* disperses primarily over short distances, short‐term recoveries in areas where local populations have been extirpated is unlikely without restoration interventions. Potential restoration efforts would therefore need to be targeted and conducted at local scales. *Postelsia* has been successfully transplanted via several methods (Nielsen et al., 2006; Paine, 1979; Paine, 1988; Paine et al., 2017), and transplanted *Postelsia* individuals have established local patches that persist for 5+ generations (Paine et al., 2017). Given the kelp's narrow habitat requirements (Nielsen et al., 2006; Paine, 1988), careful site selection for restoration activities is essential for restoration success. Active clearing of mussels and other competitors from sites with transplanted or remnant populations may also improve restoration outcomes (Paine, 1979; Paine, 1988).

Critically, Indigenous peoples have harvested *Postelsia* for millennia, so any regulatory changes or conservation and restoration efforts should be developed in conversation and partnership with tribal communities and First Nations to support co‐management and protect culturally significant practices (Ainis et al., 2019; Turner, 2001; Van Pelt et al., 2017).

### Importance of long‐term, broad‐scale ecosystem monitoring

Long‐term, broad‐scale ecological experiments and monitoring studies provide critical information about how global change influences biological communities and ecosystems (Hughes et al., 2017). These studies are increasingly important as anthropogenic stressors increase in diversity and intensity, often leading to interactive and cumulative emergent effects (Crain et al., 2008). In this study, we leverage data from 480 field surveys conducted from 1999 to 2024 at 17 field sites spanning over 1200 km of coastline to show how converging stressors were associated with a ~ 50% decline in *Postelsia* populations across more than half of the species' geographic range. Studies spanning such broad spatial and temporal scales are rare and therefore provide uniquely valuable insights into ecological and global change processes (Hughes et al., 2017).

Parsing long‐term changes from stochastic ecological dynamics is less feasible for short‐term studies or studies assessing population shifts at temporal snapshots separated by long periods (e.g., Csordas et al., 2024). In addition, many long‐term monitoring studies solely examine population dynamics of single species instead of monitoring change across multiple taxa at varying trophic levels, thereby limiting inferences into the drivers of population changes (Coletti et al., 2016). Our ability to assemble multispecies monitoring datasets provided deeper understanding of the ecological processes underpinning *Postelsia* declines (Figure 2), which should hold significant weight in environmental policy and conservation and restoration decision‐making. We therefore urge strong and consistent investment in large‐scale, long‐term, multispecies monitoring programs because of the crucial ecological and management insights they provide, particularly as global change impacts intensify into the future (Hughes et al., 2017; Lindenmayer et al., 2022).

### Limitations and future directions

Our results provide compelling evidence suggesting that direct physiological stress contributed to rapid *Postelsia* declines during the MHW and that SSWD indirectly limited *Postelsia* recoveries at sites in the southern portion of the kelp's distribution. However, our analyses remain correlative and we cannot fully disentangle causality due to the lack of experimental controls. In addition, while the *Postelsia* survey dataset we present here constitutes a majority of the species' long‐term population monitoring data—particularly in our study region (but see Csordas et al., 2024 for long‐term monitoring from Vancouver Island, BC)—the transects are small in spatial extent and may not fully capture broader scale patterns of distributional change and recolonization dynamics (i.e., at scales from a rocky intertidal bench to an entire coastline). Moreover, because transect locations were selected in part based on their safe accessibility, the dataset may underrepresent more wave‐exposed habitats where *Postelsia* population dynamics could differ. Increased wave exposure likely shifts *Postelsia* tidal height distributions upward, thereby reducing spatial overlap with *Pisaster* and potentially causing the indirect effects of SSWD on *Postelsia* to vary across wave exposure gradients.

To address these gaps, future studies should pair long‐term in situ monitoring with tools for surveying *Postelsia* abundance at broader spatial scales, such as with drones and aerial imaging (Csordas et al., 2024; Garza, 2019). Experimental and modeling efforts are also needed to define critical thermal limits for *Postelsia* under realistic field conditions, to forecast *Postelsia* range contraction or distributional shifts, and to identify potential sites for targeted conservation and restoration efforts. Future work could also explore alternative *Postelsia* dispersal mechanisms, such as the transport of fertile frond fragments by waves and wind, which may facilitate long‐distance colonization more effectively than whole‐kelp rafting and revise our understanding of *Postelsia* population connectivity (Dayton, 1973; Paine, 1988; Paine et al., 2017; P. Dayton, personal communication). Finally, deeper exploration of the social and ecological roles played by *Postelsia* can provide important insights into how kelp declines and potential recoveries influence social‐ecological system dynamics.

### Conclusions

Our study provides the most comprehensive assessment to date of *Postelsia* population dynamics across a large portion of the species' range, revealing severe and widespread declines linked to the combined impacts of the 2014–2016 MHW and SSWD‐triggered *Pisaster* losses. By leveraging long‐term, broad‐scale, multispecies ecological monitoring, we demonstrate how concurrent disturbance events can interact to reshape the population dynamics of climate‐sensitive species through both direct and indirect pathways. These findings underscore the need for conservation and management strategies that account for the complex, compounding, and interactive effects of global change.

## AUTHOR CONTRIBUTIONS

**Francis D. Gerraty** contributed to conceptualization, data curation, formal analysis, investigation, software, visualization, writing – original draft, writing – review and editing. **Karah N. Cox‐Ammann** contributed to conceptualization, investigation, writing – review and editing. **Melissa A. Douglas** contributed to investigation, writing – review and editing. **Maya George** contributed to investigation, writing – review and editing. **David P. Lohse** contributed to conceptualization, data curation, formal analysis, investigation, writing – review and editing. **C. Melissa Miner** contributed to data curation, investigation, writing – original draft, writing – review and editing. **Peter T. Raimondi** contributed to conceptualization, formal analysis, funding acquisition, investigation, supervision, writing – review and editing.

## CONFLICT OF INTEREST STATEMENT

The authors declare no competing interests.

## Supporting information


Appendix S1.

## Data Availability

Data and code (Gerraty et al., 2026) are available in Zenodo at https://doi.org/10.5281/zenodo.18274746. Study site names and site coordinates are not included in the publicly available data because *Postelsia palmaeformis* is a protected species and threatened by illegal human harvest; qualified researchers may obtain georeferenced *P. palmaeformis* monitoring data by submitting a data request from the Multi‐Agency Rocky Intertidal Network at https://www.pacificrockyintertidal.org.
