# Supplementary material for: Marine heatwave and keystone predator loss drive broad‐scale decline and hinder recovery of a rocky intertidal kelp
Source: Ecol Appl. 2026 Mar 16;36(2):e70215. doi: 10.1002/eap.70215 (PMC12991965; doi:10.1002/eap.70215)
Supplement: Supplementary file 1 — Appendix S1. [file EAP-36-e70215-s001.pdf]

ECOLOGICAL APPLICATIONS

Appendix S1

Marine heatwave and keystone predator loss drive broad-scale decline and hinder recovery of a rocky intertidal kelp

Francis D. Gerraty, Karah N. Cox-Ammann, Melissa A. Douglas, Maya George, David P. Lohse, C. Melissa Miner , Peter T. Raimondi

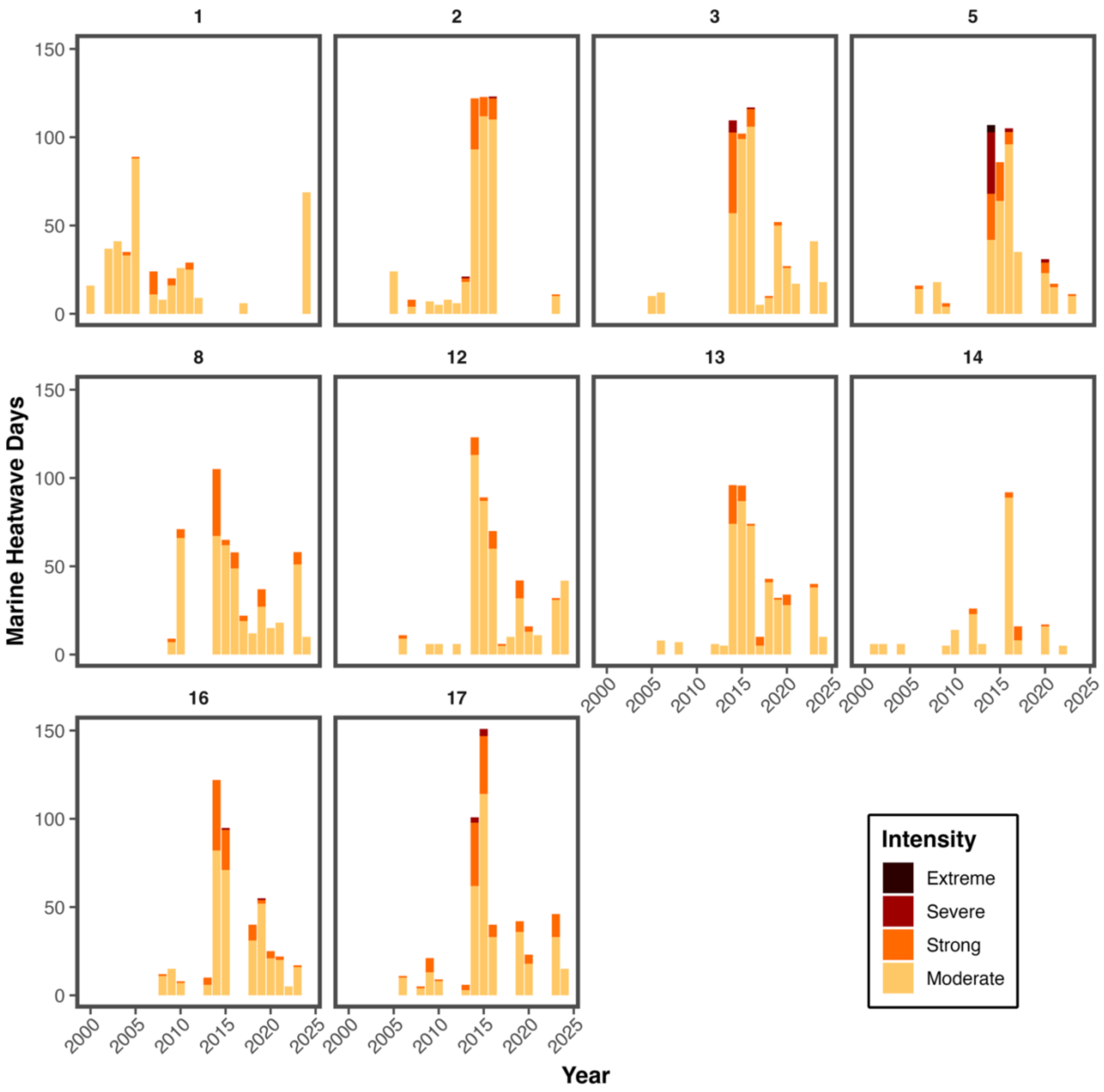

Figure S1. Number of days per year across all sites that fell within each MHW category. MHWs were defined as warm water periods lasting ≥5 days above the 90th percentile of long-term local climatology and categorized by the magnitude above this threshold: "moderate" (1-2×), "strong" (2-3×), "severe" (3-4×), and "extreme" (>4×). Note that gaps in water temperature data reflect variation in site visit frequency and temperature logger failure, malfunction, and loss.

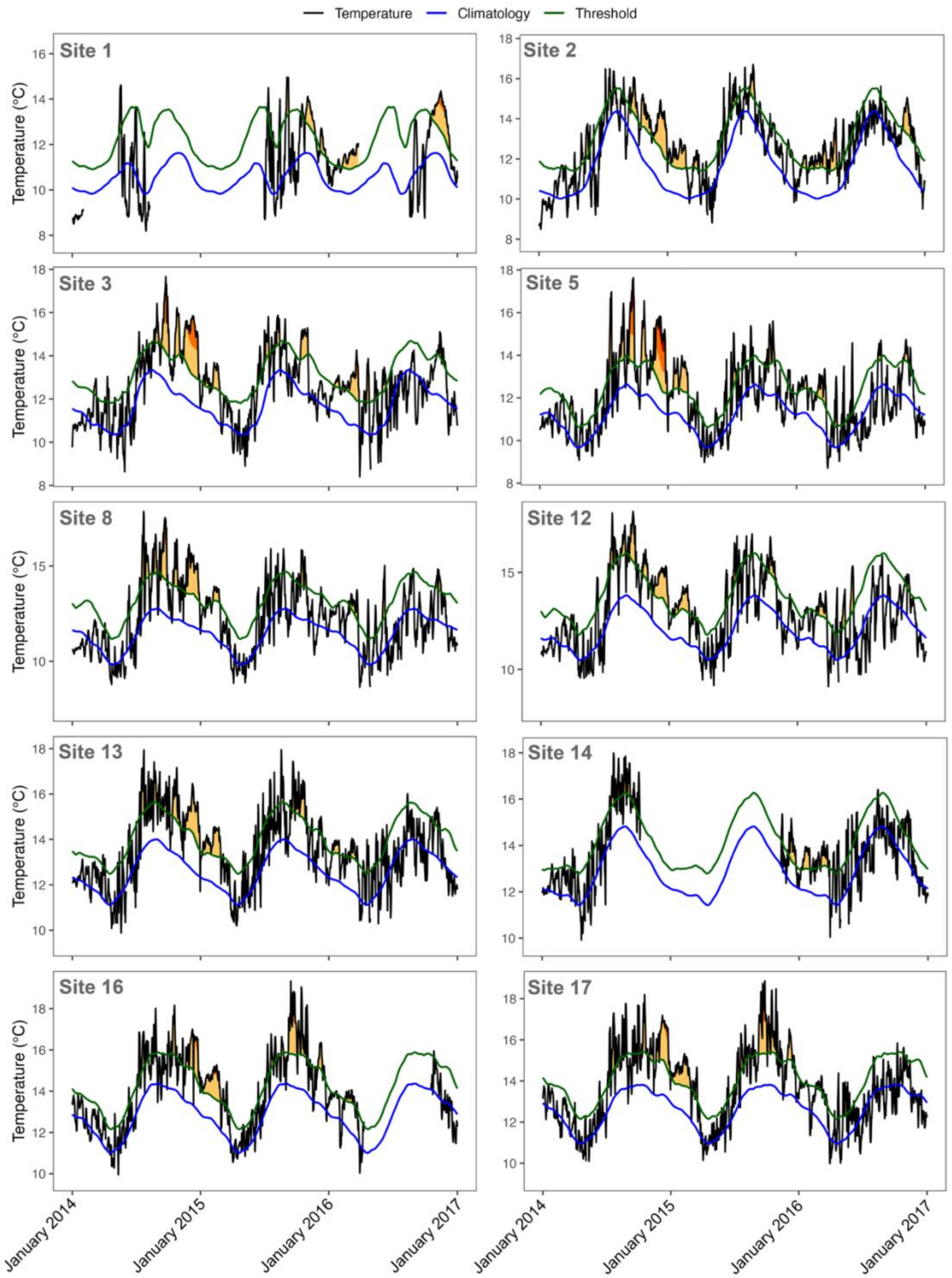

Figure S2. Intertidal water temperatures measured in rocky intertidal zones during the 2014-2016 MHW at all study sites with water temperature loggers. When water temperatures (black line) exceeded the 90th percentile (green solid line) relative to the local long-term climatology (blue line) for five or more days, then MHW events were detected (colored fills). Note that some sites did not have water temperature data due to due to temperature logger failure, malfunction, and loss.

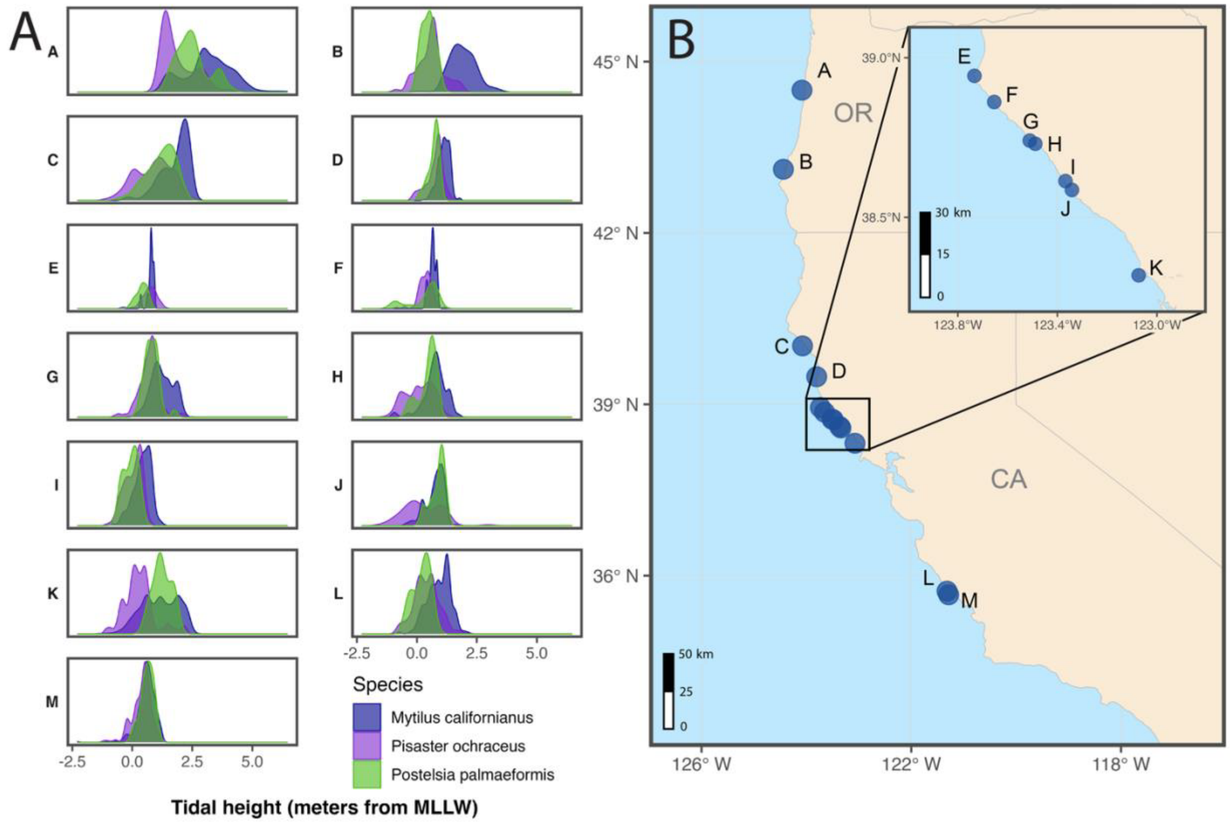

Figure S3. (A) Tidal height distributions of *Mytilus californianus*, *Pisaster*, and *Postelsia* from 44 CBS surveys conducted between 2001 and 2023 in which the three species co-occurred, across **(B)** 13 MARINe sites. Note that the sites used to examine tidal height distributions do not perfectly overlap with those used to assess *Postelsia* population changes.

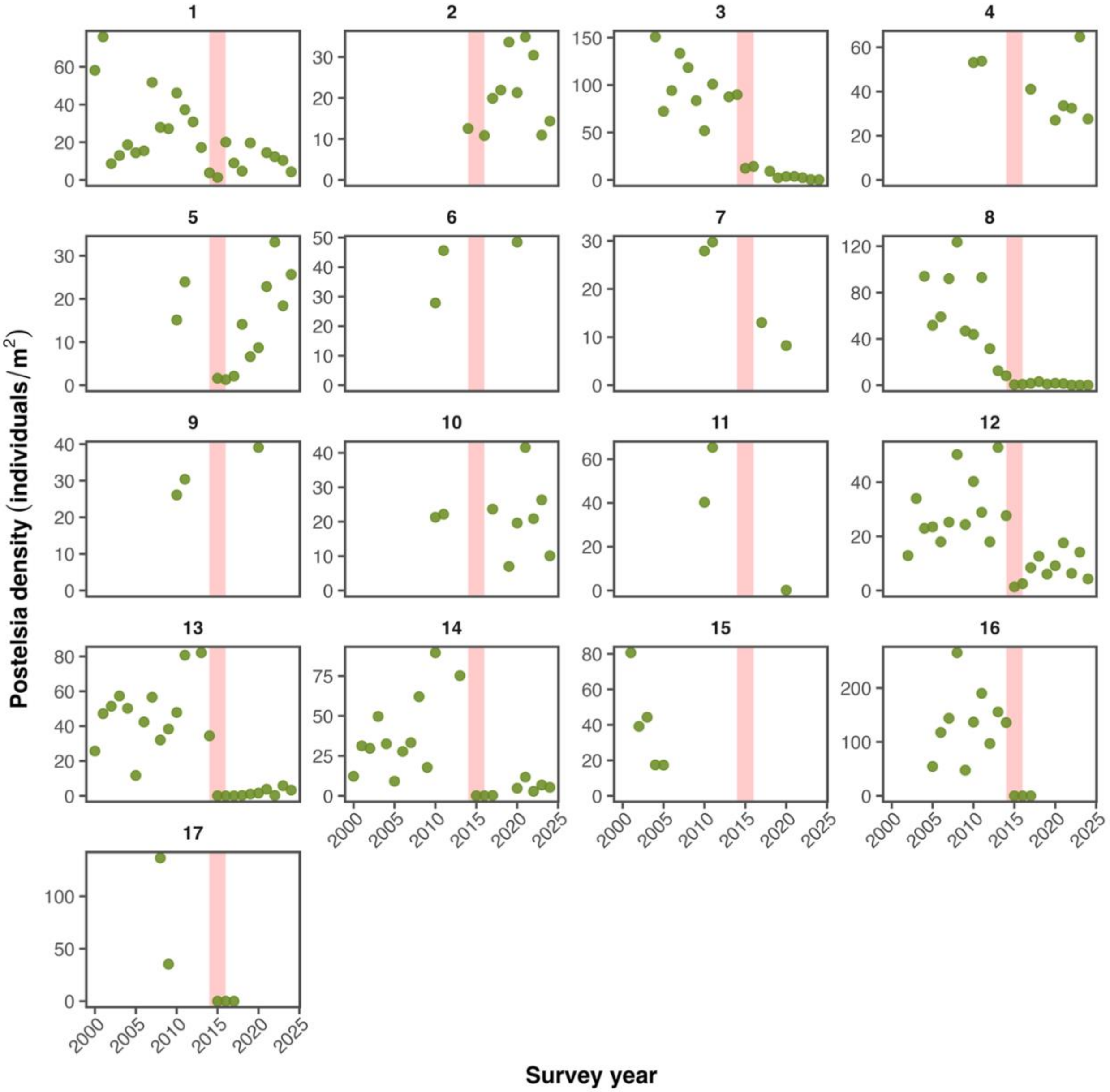

Figure S4. *Postelsia* density (# individuals/m^2) in all surveys at all sites. Shaded pink region represents the 2014-2016 MHW.

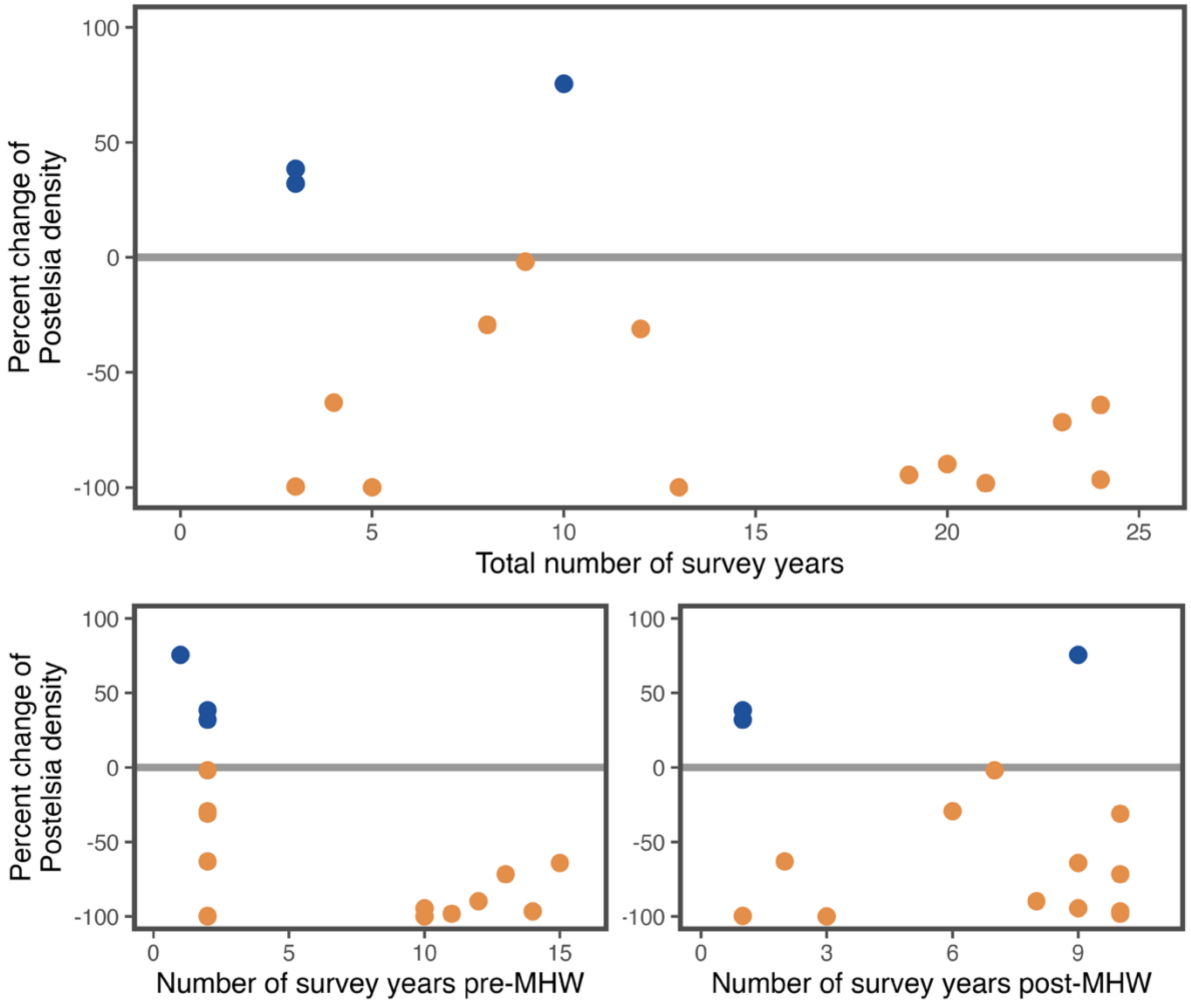

Figure S5. Percent change in mean *Postelsia* densities before (≤ 2014) versus during and after (≥ 2015) the MHW relative to **(A)** the total number of survey years, **(B)** the number of survey years pre-MHW, and **(C)** the number of survey years post-MHW (≥ 2015). Orange points indicate post-MHW declines in *Postelsia* density and blue points indicate post-MHW density increases.

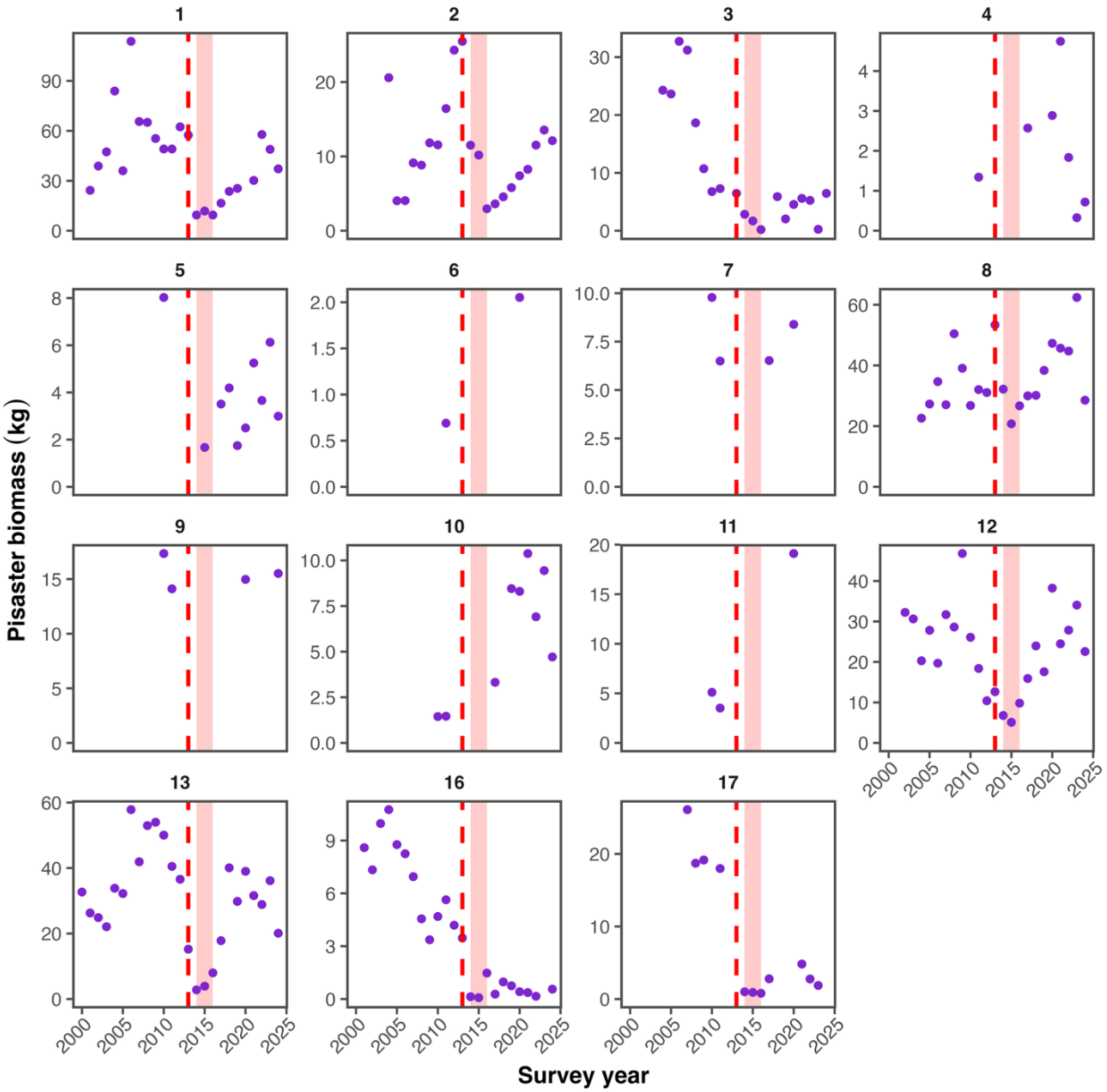

Figure S6. *Pisaster* biomass (kg) from sea star monitoring plots in all surveys at all sites. The vertical red dashed line represents the onset of the SSWD outbreak and the shaded pink region represents the 2014-2016 MHW. Note that survey area differed between sites, and we purposefully did not calculate *Pisaster* densities due to differences in habitat availability and intertidal features between sites.

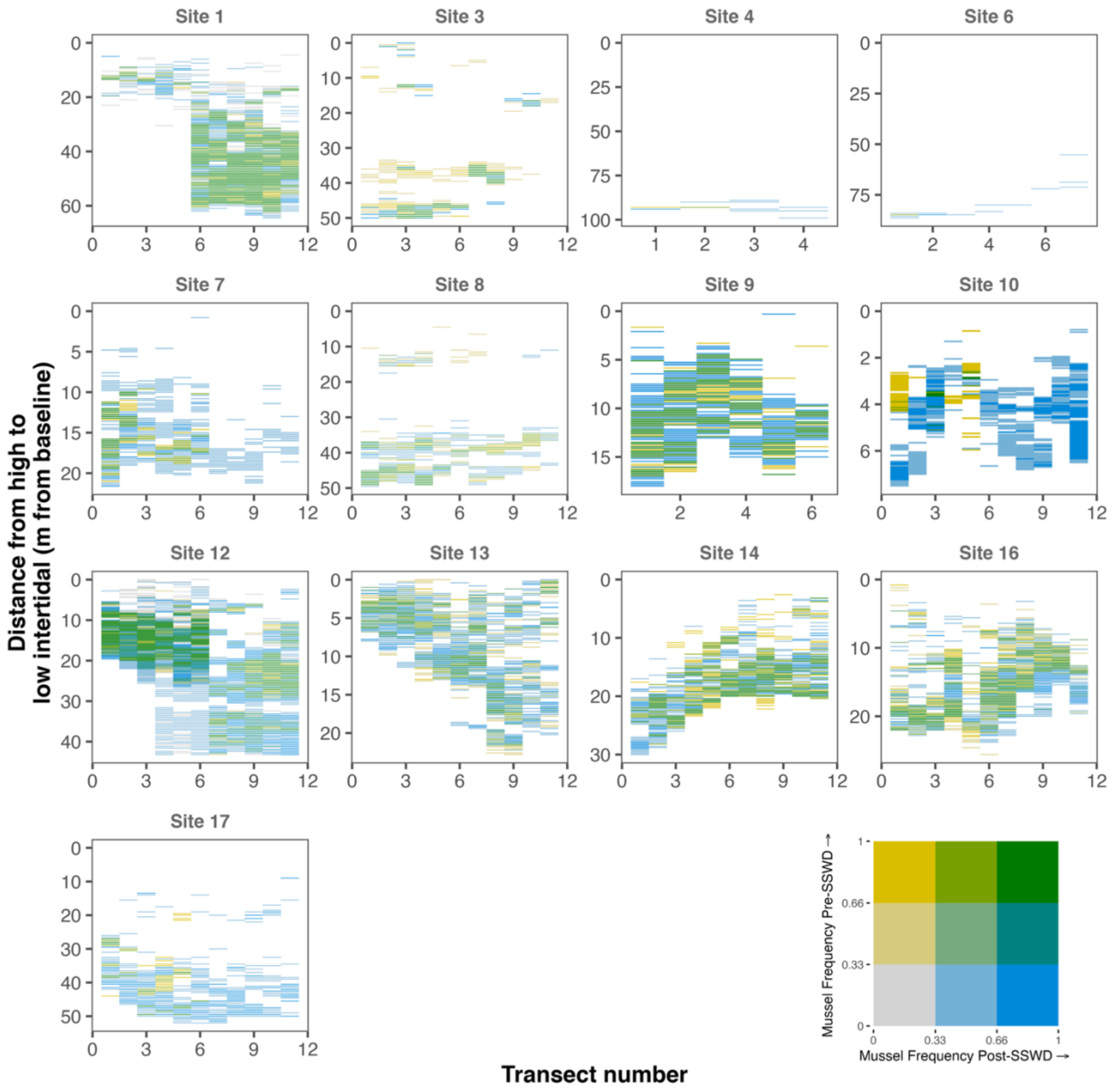

Figure S7. Presence of mussels (*M. californianus*) at replicate CBS transects (x axis) along a high-to-low (y axis) rocky intertidal gradient. Bands are colored using a bivariate palette showing mussel occurrence frequency (proportion of surveys in which mussels were detected at any single CBS survey point) before (≤ 2014; yellow scale) and after (blue scale) the SSWD outbreak. Yellow bands indicate high pre-SSWD and low post-SSWD mussel occurrence, blue bands indicate low pre-SSWD and high post-SSWD occurrence, and green bands indicate high occurrence both before and after. Uncolored points represent locations where mussels were never detected.

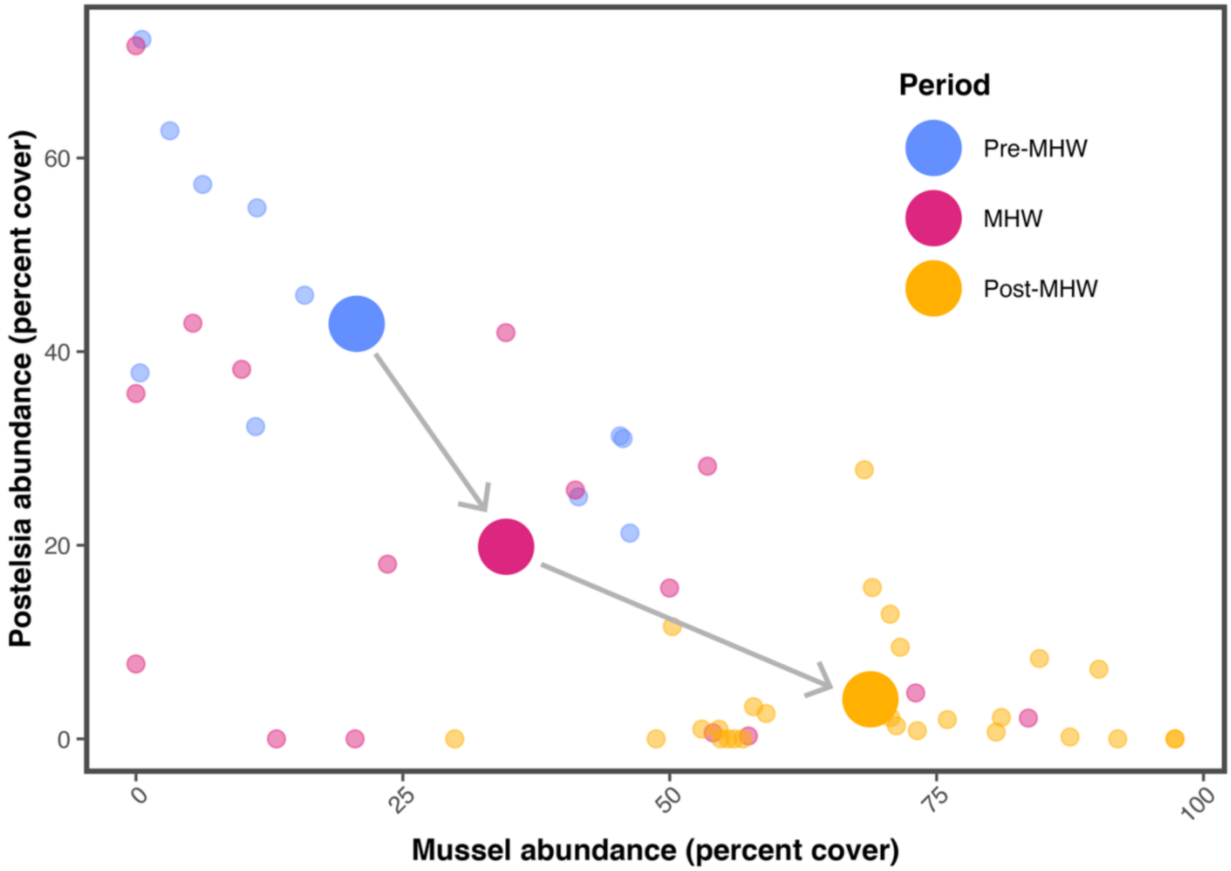

Figure S8. Temporal changes in the percent cover of *Postelsia* and mussels (*M. californianus*) within standardized boxes in repeated panoramic photos across three periods: Pre-MHW (≤ 2013), MHW (2014-2016), and Post-MHW (≥ 2017). Small points represent individual photos; large points show mean values for each period.

Table S1. Site-level summarization of rocky intertidal water temperature data.

Site-Level Summary of Water Temperature Data									
Site	# days with water temperature data	# years with water temperature data	Maximum recorded water temperature	# days with water temperature exceeding 15 C	# days with water temperature exceeding 18 C	# days classified as moderate MHW (% of days)	# days classified as strong MHW (% of days)	# days classified as severe MHW (% of days)	# days classified as extreme MHW(% of days)
1	7235	24	16.94	70	0	673.53 (9.31%)	51.11 (0.71%)	0 (0%)	0 (0%)
2	4720	17	17.70	220	0	444.09 (9.41%)	58.72 (1.24%)	2.15 (0.05%)	0 (0%)
3	6218	20	17.66	140	0	456.95 (7.35%)	62.6 (1.01%)	7.81 (0.13%)	0 (0%)
5	4535	16	17.63	76	0	351.83 (7.76%)	67.94 (1.5%)	38.86 (0.86%)	4.02 (0.09%)
8	6529	20	17.86	150	0	421.01 (6.45%)	78.92 (1.21%)	0 (0%)	0 (0%)
12	5833	19	18.24	357	4	430.9 (7.39%)	39.12 (0.67%)	0 (0%)	0 (0%)
13	8093	24	18.11	485	2	441.91 (5.46%)	47.9 (0.59%)	0 (0%)	0 (0%)
14	7569	25	20.04	771	7	403.06 (5.33%)	38.82 (0.51%)	0 (0%)	0 (0%)
16	6064	19	19.34	601	14	368.03 (6.07%)	88.62 (1.46%)	2.04 (0.03%)	0 (0%)
17	6134	20	18.86	414	11	374.99 (6.11%)	115.67 (1.89%)	7.2 (0.12%)	0 (0%)

Table S2. *Postelsia* density change at each site before (≤ 2014) versus during and after (≥ 2015) the MHW.

Site-Level Summary of Postelsia Density Data

Site	Georegion	Total survey years	# survey years pre-MHW	# survey years post-MHW	Density pre-MHW (+/- SE)	Density post-MHW (+/- SE)	Percent change in density
1	OR	24	15	9	29.72 (+/- 5.29)	10.66 (+/- 2.21)	-64.128448
2	CA North	10	1	9	12.55	22.02 (+/- 3.08)	75.498008
3	CA North	19	10	9	98.29 (+/- 9.22)	5.38 (+/- 1.74)	-94.524894
4	CA North	8	2	6	53.42 (+/- 0.31)	37.75 (+/- 5.78)	-29.339885
5	CA North	12	2	10	19.52 (+/- 4.42)	13.45 (+/- 3.55)	-31.088203
6	CA North	3	2	1	36.72 (+/- 8.87)	48.47	32.001816
7	CA North	4	2	2	28.81 (+/- 0.93)	10.63 (+/- 2.39)	-63.105111
8	CA North	21	11	10	59.66 (+/- 11.10)	1.08 (+/- 0.30)	-98.191111
9	CA North	3	2	1	28.25 (+/- 2.15)	39.09	38.358255
10	CA North	9	2	7	21.73 (+/- 0.44)	21.31 (+/- 4.30)	-1.931242
11	CA North	3	2	1	52.80 (+/- 12.54)	0.17	-99.670646
12	CA North	23	13	10	29.15 (+/- 3.38)	8.26 (+/- 1.66)	-71.648840
13	CA Central	24	14	10	47.00 (+/- 5.12)	1.61 (+/- 0.64)	-96.569149
14	CA Central	20	12	8	39.19 (+/- 7.24)	4.00 (+/- 1.44)	-89.801965
15	CA Central	5	5	0	39.76 (+/- 11.61)	NA	NA
16	CA Central	13	10	3	134.42 (+/- 20.12)	0.00 (+/- 0.00)	-100.000000
17	CA Central	5	2	3	85.84 (+/- 50.56)	0.01 (+/- 0.01)	-99.992809
